# Supplementary material for: Zero-sum beliefs and the avoidance of political conversations
Source: Commun Psychol. 2024 May 16;2:43. doi: 10.1038/s44271-024-00095-4 (PMC11332089; doi:10.1038/s44271-024-00095-4)
Supplement: Supplementary file 1 — Supplemental Materials [file 44271_2024_95_MOESM1_ESM.pdf]

Supplementary Information for “Zero-Sum Beliefs and the Avoidance of Political Conversations”

Contents

Measures used …………………………………………………………………………………1

Supplementary Note …………………………………………………………………………………3

Measures Used

Zero-Sum Beliefs about Politics (Studies 1 and 2)

- When one political party gains it inevitably comes at another party's expense
- When one political party succeeds it means that another political party fails
- If a political party wants to succeed, it has to do so at another party's expense
- When lawmakers from one party pass a bill, it comes at the expense of voters of other political parties
- Politics is not a zero-sum game
- When one political party gains, other political parties can also gain

Receptiveness to Opposing Views (Studies 1 and 2)

- I am willing to have conversations with individuals who hold strong views opposite to my own
- I like reading well thought-out information and arguments supporting viewpoints opposite to mine
- I find listening to opposing views informative
- I value interactions with people who hold strong views opposite to mine
- I am generally curious to find out why other people have different opinions than I do
- People who have opinions that are opposite to mine often have views which are too extreme to be taken seriously
- People who have views that oppose mine rarely present compelling arguments
- Information from people who have strong opinions that oppose mine is often designed to mislead less-informed listeners
- Some points of view are too offensive to be equally represented in the media
- Some issues are just not up for debate
- Some ideas are simply too dangerous to be part of public discourse
- I consider my views on some issues to be sacred
- People who have views that oppose mine are often biased by what would be best for them and their group
- People who have views that oppose mine often base their arguments on emotion rather than logic
- Listening to people with views that strongly oppose mine tends to make me angry
- I feel disgusted by some of the things that people with views that oppose mine say
- I often feel frustrated when I listen to people with social and political views that oppose mine
- I often get annoyed during discussions with people with views that are very different from mine

Perceived Conflict (Studies 1 and 2)

- Talking about politics always creates harmful conflict between neighbors, friends, and family members
- Talking about politics creates friction between neighbors, friends, and family members
- Talking about politics creates animosity between neighbors, friends, and family members
- Talking about politics pits neighbors, friends, and family members against each other
- Talking about politics puts tension on relationships between neighbors, friends, and family members

Avoidance of Political Conversations (Study 1 and Study 2, Time 1)

- In the past month I have avoided talking politics with family members with whom I disagree
- In the past month I have avoided talking politics with friends with whom I disagree
- In the past month I have avoided talking politics with strangers with whom I disagree
- In the past month I have avoided talking politics with neighbors with whom I disagree

Avoidance of Political Conversations (**Study 2, Time 2**)

- In the past week I have avoided talking politics with family members with whom I disagree
- In the past week I have avoided talking politics with friends with whom I disagree
- In the past week I have avoided talking politics with strangers with whom I disagree
- In the past week I have avoided talking politics with neighbors with whom I disagree

Supplementary Note 1

World Values Survey Results by Country 2017-2022, retrieved from *https://www.worldvaluessurvey.org/WVSDocumentationWV7.jsp (June 14, 2023)*

World Values Survey Wave 7 (2017-2022)
Results in % by country weighted by w_weight

v5.0

Q200- When you get together with your friends, would you say you discuss political matters frequently, occasionally or never?

	TOTAL	How often discusses political matters with friends					
		Frequently	Occasionally	Never	Don't know	No answer	Missing Unknown
Andorra	1,004	13.0	54.9	29.7	-	2.4	-
Argentina	1,003	10.3	49.1	39.4	1.1	0.2	-
Australia	1,813	11.3	62.8	25.2	-	0.7	-
Bangladesh	1,200	10.2	43.2	43.2	2.8	0.6	-
Armenia	1,223	44.4	36.4	18.7	0.1	0.4	-
Bolivia	2,067	6.8	46.1	46.8	0.3	-	-
Brazil	1,762	11.8	47.0	39.8	1.1	0.3	-
Myanmar	1,200	4.2	49.9	45.8	-	-	-
Canada	4,018	16.4	61.5	22.0	-	-	-
Chile	1,000	4.3	42.4	51.5	0.7	1.1	-
China	3,036	7.2	56.2	36.4	-	0.2	-
Taiwan ROC	1,223	4.4	45.5	50.1	-	-	-
Colombia	1,520	12.1	35.7	52.2	-	-	-
Cyprus	1,000	13.3	55.2	30.6	0.2	0.6	-
Czechia	1,200	6.2	66.4	27.3	0.1	0.1	-
Ecuador	1,200	9.5	38.6	51.5	0.2	-	0.2
Ethiopia	1,230	9.6	57.9	30.7	1.7	0.1	-
Germany	1,528	24.9	62.6	12.6	-	-	-
Greece	1,200	16.1	56.4	26.6	0.6	0.3	-
Guatemala	1,229	8.8	63.1	26.6	-	1.5	-
Hong Kong SAR	2,075	5.0	63.8	31.1	-	0.1	-
Indonesia	3,200	6.7	43.7	49.5	0.0	-	-
Iran	1,499	16.4	50.2	33.3	-	0.1	-
Iraq	1,200	9.9	42.7	44.1	2.1	1.2	-
Japan	1,353	3.5	47.7	46.3	2.0	0.6	-
Kazakhstan	1,276	7.8	58.4	30.8	0.9	2.1	-
Jordan	1,203	11.2	37.0	50.2	1.6	-	-
Kenya	1,266	18.2	51.1	28.4	1.5	0.8	-
South Korea	1,245	9.6	66.7	23.8	-	-	-
Kyrgyzstan	1,200	10.3	48.4	41.0	0.2	0.1	-
Lebanon	1,200	16.5	53.0	30.5	-	-	-
Libya	1,196	19.9	47.7	31.6	0.6	0.2	0.1
Macau SAR	1,023	6.5	71.4	21.9	-	-	0.2
Malaysia	1,313	12.0	68.8	19.2	-	-	-
Maldives	1,039	15.2	54.5	30.1	-	-	0.2
Mexico	1,741	7.5	44.8	47.7	-	-	-
Mongolia	1,638	8.6	66.6	23.7	1.1	-	-
Morocco	1,200	16.3	55.2	28.4	-	-	-
Netherlands	2,145	11.8	62.3	18.2	1.5	0.7	5.5
New Zealand	1,057	15.6	67.8	14.7	-	-	1.9
Nicaragua	1,200	62.3	30.2	7.5	-	-	-
Nigeria	1,237	21.0	50.4	28.0	0.4	0.2	-
Pakistan	1,995	17.9	48.5	32.3	1.1	0.2	-
Peru	1,400	8.6	52.4	38.8	0.2	0.0	-
Philippines	1,200	11.7	71.8	16.5	-	-	-
Puerto Rico	1,127	8.2	34.3	56.4	-	1.2	-
Romania	1,257	7.2	48.5	42.4	1.5	0.4	-
Russia	1,810	10.4	56.0	31.5	2.0	0.1	-
Serbia	1,046	8.5	53.5	37.6	0.2	0.2	-
Singapore	2,012	7.1	54.7	37.7	-	0.5	-
Slovakia	1,200	6.8	59.3	33.7	-	0.2	-
Vietnam	1,200	2.9	51.2	45.8	-	-	-
Zimbabwe	1,215	8.6	44.8	46.2	0.2	0.2	0.2
Tajikistan	1,200	10.2	48.6	41.2	-	-	-
Thailand	1,500	11.7	59.1	28.7	-	0.5	-
Tunisia	1,208	9.9	45.3	42.8	0.2	0.2	1.7
Turkey	2,415	8.2	60.0	30.1	0.8	0.9	0.0
Ukraine	1,289	7.8	58.9	29.2	3.8	0.3	-

Page 562 of 910

	TOTAL	How often discusses political matters with friends					
		Frequently	Occasionally	Never	Don't know	No answer	Missing Unknown
Egypt	1,200	3.8	41.5	54.2	0.6	-	-
Great Britain	2,609	18.1	58.2	23.5	0.1	0.1	0.0
United States	2,596	68.2	20.8	9.5	0.1	1.4	-
Uruguay	1,000	17.3	29.5	52.7	0.4	0.1	-
Venezuela	1,190	13.9	53.8	32.4	-	-	-
Northern Ireland	447	17.8	51.4	30.7	-	0.1	-
**TOTAL**	(94,278)	13.6%	52.1%	33.4%	0.5%	0.3%	0.2%
