# Supplementary material for: Zero-sum beliefs and the avoidance of political conversations
Source: Commun Psychol. 2024 May 16;2:43. doi: 10.1038/s44271-024-00095-4 (PMC11332089; doi:10.1038/s44271-024-00095-4)
Supplement: Supplementary file 3 — Reporting Summary [file 44271_2024_95_MOESM3_ESM.pdf]

nature portfolio

Corresponding author(s): Shai Davidai

Last updated by author(s): 04.19.24

Reporting Summary

Nature Portfolio wishes to improve the reproducibility of the work that we publish. This form provides structure for consistency and transparency in reporting. For further information on Nature Portfolio policies, see our Editorial Policies and the Editorial Policy Checklist.

Statistics

For all statistical analyses, confirm that the following items are present in the figure legend, table legend, main text, or Methods section.

n/a	Confirmed	
☐	☒	The exact sample size (*n*) for each experimental group/condition, given as a discrete number and unit of measurement
☐	☒	A statement on whether measurements were taken from distinct samples or whether the same sample was measured repeatedly
☐	☒	The statistical test(s) used AND whether they are one- or two-sided<br>*Only common tests should be described solely by name; describe more complex techniques in the Methods section.*
☐	☒	A description of all covariates tested
☐	☒	A description of any assumptions or corrections, such as tests of normality and adjustment for multiple comparisons
☐	☒	A full description of the statistical parameters including central tendency (e.g. means) or other basic estimates (e.g. regression coefficient) AND variation (e.g. standard deviation) or associated estimates of uncertainty (e.g. confidence intervals)
☐	☒	For null hypothesis testing, the test statistic (e.g. *F*, *t*, *r*) with confidence intervals, effect sizes, degrees of freedom and *P* value noted<br>*Give P values as exact values whenever suitable.*
☒	☐	For Bayesian analysis, information on the choice of priors and Markov chain Monte Carlo settings
☒	☐	For hierarchical and complex designs, identification of the appropriate level for tests and full reporting of outcomes
☐	☒	Estimates of effect sizes (e.g. Cohen's *d*, Pearson's *r*), indicating how they were calculated

Our web collection on statistics for biologists contains articles on many of the points above.

Software and code

Policy information about availability of computer code

Data collection	n/a
Data analysis	n/a

For manuscripts utilizing custom algorithms or software that are central to the research but not yet described in published literature, software must be made available to editors and reviewers. We strongly encourage code deposition in a community repository (e.g. GitHub). See the Nature Portfolio guidelines for submitting code & software for further information.

Data

Policy information about availability of data

All manuscripts must include a data availability statement. This statement should provide the following information, where applicable:

- Accession codes, unique identifiers, or web links for publicly available datasets
- A description of any restrictions on data availability
- For clinical datasets or third party data, please ensure that the statement adheres to our policy

Materials and data can be found at: https://osf.io/fmber/?view_only=c0f1d24f4b544af59e64eebf83ce5e88

(this statements also appears verbatim in the manuscript)

Human research participants

Policy information about studies involving human research participants and Sex and Gender in Research.

Reporting on sex and gender	Gender based information was collected as self-reported and is reported in the methods section in the manuscript. The reported findings were not moderated by gender.
Population characteristics	see below.
Recruitment	Participants were recruited via two sources. For Study 1, participants were recruited through Midgam - an Israeli web-based research panel. For Study 2, participants were recruited via Prolific Academic. Participants were told that they would take part in a general study about the views and attitudes about social topics and completed an informed consent form prior to beginning their participation.
Ethics oversight	The studies were approved by the ethics board at Columbia University.

Note that full information on the approval of the study protocol must also be provided in the manuscript.

Field-specific reporting

Please select the one below that is the best fit for your research. If you are not sure, read the appropriate sections before making your selection.

☐ Life sciences ☒ Behavioural & social sciences ☐ Ecological, evolutionary & environmental sciences

For a reference copy of the document with all sections, see nature.com/documents/nr-reporting-summary-flat.pdf

Behavioural & social sciences study design

All studies must disclose on these points even when the disclosure is negative.

Study description	All studies reported in the manuscript are quantitative, using correlational methods.
Research sample	The sample sources are described above, under "Human research participants." Although the sample of participants skews a bit younger than the general population, previous research has shown that responses collected on these sample populations are equivalent to responses collected on other samples, both online and in person. All demographic information, including age, gender, and racial/ethnic breakdown, are provided in the manuscript.
Sampling strategy	Sample size were determined in advance for each study keeping in minds the availability of funds. The manuscript reports sensitivity analyses for all studies, computing the minimum effect size that can be detected with 80% power given each sample size.
Data collection	Data collection in all studies was conducted online. Participants were directed from Midgam (Study 1) and Academic Prolific (Study 2) to an external survey hosted by Qualtrics. Participation in all studies was anonymous and voluntary.
Timing	All studies were collected on November, 2022.
Data exclusions	19 participants who failed a simple attention check were excluded from Study 2.
Non-participation	All studies were conducted online and therefore there was no tracking of drop out' rate
Randomization	The studies were correlational, and thus no randomization was needed.

Reporting for specific materials, systems and methods

We require information from authors about some types of materials, experimental systems and methods used in many studies. Here, indicate whether each material, system or method listed is relevant to your study. If you are not sure if a list item applies to your research, read the appropriate section before selecting a response.

Materials & experimental systems

n/a	Involved in the study
☒	☐ Antibodies
☒	☐ Eukaryotic cell lines
☒	☐ Palaeontology and archaeology
☒	☐ Animals and other organisms
☒	☐ Clinical data
☒	☐ Dual use research of concern

Methods

n/a	Involved in the study
☒	☐ ChIP-seq
☒	☐ Flow cytometry
☒	☐ MRI-based neuroimaging
